# Evaluation of Intravenous Infusion of Ibuprofen with Paracetamol-Morphine in Pain and Satisfaction of Patients Undergoing Supratentorial Brain Surgery

**DOI:** 10.5812/aapm-139758

**Published:** 2023-11-06

**Authors:** Sohrab Salimi, Mehrdad Taheri, Hamid Reza Khayat Kashani, Farnazsadat Ghani, Faranak Behnaz, Mahshid Ghasemi

**Affiliations:** 1 Department of Anesthesiology, Anesthesiology Research Center, Imam Hossein Hospital, School of Medicine, Shahid Beheshti University of Medical Sciences, Tehran, Iran; 2 Department of Anesthesiology, Imam Hossein Hospital, School of Medicine, Shahid Beheshti University of Medical Sciences, Tehran, Iran; 3 Department of Neurosurgery, Imam Hossein Hospital, School of Medicine,Shahid Beheshti University of Medical Sciences, Tehran, Iran; 4 Anesthesiology Research Center, Imam Hossein Hospital, Shahid Beheshti University of Medical Sciences, Tehran, Iran; 5 Department of Anesthesiology, Shohada-e-Tajrish Hospital, School of Medicine, Shahid Beheshti University of Medical Sciences, Tehran, Iran; 6 Department of Anesthesiology, Akhtar Hospital, School of Medicine, Shahid Beheshti University of Medical Sciences, Tehran, Iran

**Keywords:** Ibuprofen, Paracetamol, Morphine, Supratentorial Neoplasms, Platelet Function Tests, Pain, Postoperative, Surgery

## Abstract

**Background:**

The pain experienced following supratentorial brain surgery is usually defined as moderate to severe. Therefore, pain-management approaches, including narcotics, are an integral part of treatment regimens that cause respiratory complications or seizures, and reducing this pain level and increasing patient satisfaction is vital.

**Methods:**

This randomized, double-blind clinical trial study to evaluate the pain level and satisfaction in patients undergoing surgery for supratentorial brain neoplasms was performed on two groups with a sample size of 50 patients. In group I, after removal of the brain lesion (at the beginning of dura closure), 400 mg of ibuprofen solution was infused intravenously over 30 minutes. In group II, morphine 0.07 mg/kg intravenously with 1000 mg paracetamol was infused over 30 minutes. After injecting ibuprofen and paracetamol morphine, the patient's pain level and satisfaction with the process were checked.

**Results:**

Patients' satisfaction score in the first 6 hours in the ibuprofen group was 1.67 ± 0.72, and in the other group was 2.27 ± 0.7, which was statistically different (P-value = 0.029). The mean of VAS in the first, second, third, and fourth hours was not statistically different. In the comparative analysis of the laboratory indicators of platelet function analysis in the two groups, none of the measured items had a significant difference between the two groups in the three measurement periods (P > 0.05).

**Conclusions:**

Administration of ibuprofen led to pain relief and patient satisfaction comparable to morphine and paracetamol, and after the surgery for supratentorial brain tumors, ibuprofen did not affect the patients’ blood clotting functions.

## 1. Background

The pain following supratentorial brain surgery is usually graded as moderate to severe. This level of pain intensity makes the use of reducing drugs inevitable. To reduce pain, opioids are now an integral part of treatment regimens that are associated with respiratory failure. Therefore, finding methods to reduce these risks has been the focus of much research. Pain is an unpleasant emotional experience that can be related to thermal, mechanical, or chemical tissue damage (1). Acute postoperative pain is one of the most important complications of all surgeries, which can increase mortality (2). Incomplete control of acute postoperative pain is also an important predictor of chronic postoperative pain in the long term (1). The pain experienced following supratentorial brain surgery is usually graded as moderate to severe, and its intensity makes the use of pain-reducing drugs inevitable. The use of painkillers is common during the first seven days after surgery and decreases over the next three months (3).

## 2. Methods

After being approved by the Ethics Committee of Shahid Beheshti University of Medical Sciences with the code (IR.SBMU.MSP.REC.1398.307), the study was conducted according to the Helsinki criteria, and informed consent was obtained from all patients before entering the study. The clinical trial has been registered with the identifier IRCT20210506051200N1.

Sample size calculation was performed using the results of a previous pilot study on 20 cases (10 random cases from each group), assuming an α-error of 0.05, a power of 80%, an estimated difference between the two groups as 0.487, and a standard error defined as 1.350. As a result, 50 cases were considered to be included.

A randomized controlled trial study was performed on 50 patients undergoing elective craniotomy surgery aged 18 - 65 years and ASA 1,2 physical health status. The patients were selected and randomly divided into 2 groups of 25 subjects, I (B) and II (MP). In group I, 400 mg of ibuprofen solution was infused intravenously over 30 minutes after removal of the brain lesion (at the beginning of dura closure). Additionally, in group II, morphine was infused 0.05 mg/kg intravenously with 1000 mg paracetamol for 30 minutes.

In both groups, after the patients entered the operating room after history taking, reviewing the records, and ensuring that they met the inclusion criteria with no applicable exclusion criteria, an arterial line from the non-dominant hand of the patient was obtained. Midazolam 0.02 mg/kg, fentanyl 5 μg/Kg, atracurium 0.5 mg/kg, and the anesthetic propofol 2 mg/kg were injected under anesthesia. Anesthesia was administered under TIVA with propofol 200 μg/kg/min and fentanyl 1 μg / kg/min. To relax the brain, 3% hypertonic saline 5 mL/kg was used.

Also, to prevent and reduce cerebral edema during surgery, 0.15 mg/kg intravenous dexamethasone was used. After the surgeon removed the brain mass and controlled the bleeding at the operation site and the beginning of dura closure, a blood sample was sent for CBC, PT, PTT, Ca2 +, PFa-100, INR, fibrinogen, serum osmolality, ABG. Subsequently, in group I, 400 mg of ibuprofen solution was infused intravenously over 30 minutes. In group II, morphine 0.07 mg/kg intravenously with 1000 mg paracetamol was infused over 30 minutes. Then, half an hour and three hours after the injection of ibuprofen and morphine-paracetamol, the same tests were sent again, and the coagulation effects of these drugs were investigated in two groups.

It should be noted that the PFA-100 (platelet function analyzer) system is a platelet function analyzer designed to measure primary platelet-dependent homeostasis. Patients' pain scores and satisfaction were evaluated at 6-hour intervals (for 24 hours). The severity of pain with VAS and the level of satisfaction with a Likert scale from 0 to 5 was measured.

To measure pain, a 10 cm line marked with the words “no pain" on one end and “worst pain" on the other was used. At one end, it was “painless", and at the other end, it was “worst pain" or “indescribable pain". The patients placed a cross mark on the line to indicate the severity of the pain. A doctor then measures the line with a ruler to obtain a pain score. Also, patient’s satisfaction during the postoperative period was evaluated every 6 hours based on the following criteria: 0 for unconditionally no pain, 1 for occasionally moderate pain, 2 for continuously moderate pain, 3 for occasional severe pain, 4 for constant moderate pain and sometimes severe pain and 5 for undisrupted severe pain.

### 2.1. Statistical Analysis 

To define data, we used incidence (percent), mean ± SD, median, and range. After investigating the hypothesis of normal data, using the Shapiro test, we used an independent *t*-test to compare oxygen levels between the two groups. Chi-square tests were also used to compare qualitative variables. A P-value less than 0.05 was considered statistically significant. All statistical analyses were conducted by SPSS software (IBM Corp. Released 2016. IBM SPSS Statistics for Windows, Version 24.0. Armonk, NY: IBM Corp.).

## 3. Results

We enrolled 50 patients undergoing elective craniotomy surgery aged 18 - 65 years: 25 in the ibuprofen group and 25 in the paracetamol-morphine group. In the former, 9 (36%) were male and 16 (64%) were female, with a mean age of 41.25 ± 14.34 years, and in the latter, 13 (52%) were male and 12 (48%) were female with the mean age of 45.18 ± 13.64 years (P-value = 0.254). In addition, the duration of surgery, the patient's level of consciousness, the amount of intraoperative bleeding, and the number of units of blood received did not differ significantly between the two groups (P-value > 0.05) (Table 1). 

**Table 1.** Comparing the Basic and Clinical Characteristics of Patients in the Two Groups ^a^

Variables	Ibuprofen	Morphine + Paracetamol	P-Value
**Sex**			0.989 ^b^
Male	9 (36.0)	13 (52.0)	
Female	16 (64.0)	12 (48.0)	
**Age (y)**	41.25 ± 14.34	45.18 ± 13.64	0.989 ^c^
**GCS anesthesia**	13.20 ± 2.27	12.88 ± 0.72	0.506 ^c^
**GCS surgery**	13.28 ± 2.17	13.16 ± 0.37	0.786 ^c^
**Duration of surgery (min)**	15.88 ± 7.99	16.96 ± 7.09	0.616 ^c^
**Intraoperative bleeding**	606.00 ± 487.62	484.00 ± 243.55	0.269 ^c^

^a^ Values are expressed as No. (%) or Mean ± SD.

^b^ P-value based on chi-square.

^c^ P-value based on *t*-test.

### 3.1. Evaluation of Patients' VAS Scores at 6-Hour Intervals

Table 2 demonstrates the mean VAS scores of the patients. The mean VAS score of the patients in the first 6 hours in the ibuprofen group was 2.93 ± 1.03, and in the opposite group was 3.6 ± 0.99, which was not statistically significant (P-value = 0.081).

**Table 2.** Comparison of Mean VAS Score of Patients Between Groups

			P-Value ^a^
	Ibuprofen	Morphine	
**First 6 h**	2.93 ± 1.03	3.6 ± 0.99	0.081
**Second 6 h**	2.2 ± 0.77	2.13 ± 0.64	0.799
**Third 6 h**	1.2 ± 0.56	1.33 ± 0.49	0.493
**Fourth 6 h**	0.73 ± 0.46	0.87 ± 0.35	0.379

^a^ P-value based on *t*-test

The mean of VAS in the second, third, and fourth hours in the first group was 2.2 ± 0.77, 1.2 ± 0.56, and 0.73 ± 0.46, respectively, and in the opposite group, was 2.13 ± 0.64 and 1.33 ± 0.49 and 0.87, respectively (P-value = 0.35 which indicates no statistically significant difference).

### 3.2. Evaluation of Patients' Satisfaction Scores at 6-Hour Intervals

Table 3 tabulates the mean score of patient satisfaction. The patient's satisfaction score in the first 6 hours in the ibuprofen group was 1.67 ± 0.72, and in the opposite group was 2.27 ± 0.7, which was statistically significant (P-value = 0.029).

**Table 3.** Comparison of Mean Scores of Patient Satisfaction Between Groups

			P-Value ^a^
	Ibuprofen	Morphine	
**First 6 h**	1.67 ± 0.72	2.27 ± 0.7	0.029
**Second 6 h**	1.6 ± 0.74	1.53 ± 0.52	0.776
**Third 6 h**	0.87 ± 0.64	1.07 ± 0.26	0.271
**Fourth 6 h**	0.53 ± 0.52	0.86 ± 0.36	0.063

^a^ P-value based on *t*-test

The average satisfaction in the second, third, and fourth 6 hours in the first group was 1.6 ± 0.74 and 0.87 ± 0.64, and 0.53 ± 0.52, and in the opposite group was 1.53 ± 0.52 and 1.07 ± 0.26 and 0.86 ± 0.36, which was not statistically different.

### 3.3. Evaluation of Coagulation Status and Platelet Function

Based on the results tabulated in Table 4, which shows the comparative analysis of the laboratory indicators of the patients of the two groups, none of the measured items had a significant difference between the two groups in the three measurement periods (P > 0.05).

**Table 4.** Comparison of Laboratory Data Between Two Groups of Patients in the Three Time Periods

Indexes	Ibuprofen (n = 25)	Morphine (n = 25)	P-Value
**PT**			
Base	12.16 ± 9.23	13.16 ± 10.09	0.130
After 0.5 h	11.94 ± 9.19	14.08 ± 10.41	0.055
After 3 h	11.91 ± 9.30	12.94 ± 9.00	0.100
**PTT**			
Base	28.70 ± 16.08	25.98 ± 15.07	0.093
After 0.5 h	26.18 ± 14.91	24.12 ± 12.95	0.141
After 3 h	22.55 ± 14.27	21.88 ± 13.09	0.190
**INR**			
Base	1.15 ± 0.637	1.226 ± 0.637	0.088
After 0.5 h	1.106 ± 0.801	1.328 ± 0.834	0.072
After 3 h	1.09 ± 0.800	1.108 ± 0.813	0.079
**Hb**			
Base	11.32 ± 6.29	10.73 ± 6.830	0.090
After 0.5 h	10.92 ± 6.07	9.38 ± 6.001	0.151
After 3 h	9.08 ± 6.00	9.01 ± 6.21	0.650
**HCT**			
Base	35.11 ± 21.96	33.22 ± 19.88	0.051
After 0.5 h	34.85 ± 20.66	31.62 ± 19.705	0.190
After 3 h	33.46 ± 19.87	31.58 ± 19.00	0.058
**Plt**			
Base	190.40 ± 110.94	222.4 ± 12.91	0.066
After 0.5 h	186.61 ± 109.29	221.6 ± 126.18	0.057
After 3 h	189.25 ± 115.01	242.8 ± 129.94	0.052
**PFa**			
Base	138.61 ± 97.08	149.6 ± 99.01	0.102
After 0.5 h	142.36 ± 98.26	135.4 ± 96.88	0.220
After 3 h	144.09 ± 99.00	128.8 ± 88.06	0.090

## 4. Discussion

To evaluate the outcomes of satisfaction and VAS scores in patients with ibuprofen across paracetamol morphine, some similar studies have been checked.

In 2016, Singla et al. conducted a multi-centered, double-blind, placebo-controlled group. All 185 patients under orthopedic surgery in two groups received 800 mg IV-ibuprofen and placebo every 6 hours, and all received morphine for pain control. Similar to our study, the efficacy of IV ibuprofen was demonstrated by measuring the patients’ self-assessment of pain using VAS and verbal response scale (VRS). Pre- and post-operative administration of IV ibuprofen significantly reduced both pain and morphine use in orthopedic surgery (4).

In 2014, Moore et al. conducted a double-blind study to measure the VAS score following third molar extraction in 5 groups of patients (placebo, paracetamol, ibuprofen sodium, ibuprofen-poloxamer). Paracetamol, as a placebo, was able to lower VAS scores in a small number of patients and keep them stable. In the case of the ibuprofen formulation, VAS scores decreased rapidly during the first hour and then remained stable until re-administration. The analysis of all patients showed that the rapid reduction of VAS in the first hour relieved the pain without the need for additional analgesia within 6 hours (5).

In 2011, in a double-blinded study of patients with abdominal hysterectomy surgery, Kroll et al. measured the VAS score in those who received IV-Ibuprofen and placebo every 6 hours and reported that ambulation was significantly faster in the IV-ibuprofen treated group (6).

In our study, the VAS score in the ibuprofen groups compared to the paracetamol group in the first 6 hours was not significant. The effect of ibuprofen was weaker compared to the other group. In other studies, ibuprofen was more effective than placebo or other drugs, but in our findings, ibuprofen and paracetamol had a similar analgesic effect.

Similarly, the second, third, and fourth duration in the ibuprofen group against the morphine-paracetamol group did not have any difference, and they had equal effects.

In this study, we evaluated patient satisfaction that we had not evaluated before. According to the findings, patients in the ibuprofen group were more satisfied than the morphine-paracetamol group. The second, third, and fourth intervals were not different from the other groups.

Platelet dysfunction is a concern for postoperative pain control with NSAIDs. As a result, the risk of bleeding was investigated in this study, and no dysfunction was observed. In coagulation and platelet function tests, no major disorders were observed between the two groups, and no disorders of clotting factors and platelet function were observed in any group.

Our study demonstrated that ibuprofen was as effective as the morphine-paracetamol combination in reducing pain after supratentorial brain operations. Therefore, it is suggested that ibuprofen should be used for pain control following operations to avoid potential side effects of morphine (sedation and respiratory depression) without being concerned about platelet dysfunction or bleeding following brain surgery.

### 4.1. Conclusions

Ibuprofen was as effective as morphine and paracetamol in pain relief and patient satisfaction. After a supratentorial brain tumor operation, ibuprofen did not affect the coagulation status of the patients.

## Clinical Trial Registration Code:

IRCT20210506051200N1.
